# Following instructions in a virtual school: Does working memory play a role?

**DOI:** 10.3758/s13421-015-0579-2

**Published:** 2015-12-17

**Authors:** Agnieszka J. Jaroslawska, Susan E. Gathercole, Matthew R. Logie, Joni Holmes

**Affiliations:** MRC Cognition and Brain Sciences Unit, 15 Chaucer Road, Cambridge, CB2 7EF UK

**Keywords:** Working memory, Following instructions, Virtual environment

## Abstract

Accumulating evidence that working memory supports the ability to follow instructions has so far been restricted to experimental paradigms that have greatly simplified the practical demands of performing actions to instructions in everyday tasks. The aim of the present study was to investigate whether working memory is involved in maintaining information over the longer periods of time that are more typical of everyday situations that require performing instructions to command. Forty-two children 7–11 years of age completed assessments of working memory, a real-world following-instructions task employing 3-D objects, and two new computerized instruction-following tasks involving navigation around a virtual school to complete a sequence of practical spoken commands. One task involved performing actions in a single classroom, and the other, performing actions in multiple locations in a virtual school building. Verbal working memory was closely linked with all three following-instructions paradigms, but with greater association to the virtual than to the real-world tasks. These results indicate that verbal working memory plays a key role in following instructions over extended periods of activity.

The ability to follow instructions successfully is vital for effective cognitive functioning, in situations ranging from a child carrying out a multistep learning activity under the teacher’s guidance, a driver using spoken instructions to navigate a journey to an unfamiliar destination, or an individual following a complex medication schedule that involves differing doses and drugs. Each of these activities has been recognized to be challenging and prone to error (e.g., Gathercole & Alloway, 2008; Osterberg & Blaschke, 2005; Wickens, Toplak, & Wiesenthal, 2008). One important constraint is the capacity of working memory to retain critical information bridging the period from when instructions are being received through to their performance (e.g., Allen & Waterman, 2015; Engle, Carullo, & Collins, 1991; Gathercole, Durling, Evans, Jeffcock, & Stone, 2008; Yang, Gathercole, & Allen, 2014).

A limitation of the experimental paradigms of instruction-following to date is that they have simplified the practical demands of these real-life situations. For example, tasks have typically involved the simple manipulation of objects placed in the immediate line of vision of participants and located within an easy hand’s reach (Allen & Waterman, 2015; Engle et al., 1991; Gathercole et al., 2008; Yang, Allen, & Gathercole, 2015b; Yang, Allen, Yu, & Chan, 2015a; Yang et al., 2014). This enables even lengthy sequences of actions to be executed rapidly. In contrast, when the child in the classroom, the driver behind the wheel, or the individual taking medication is following instructions, the specific actions are often more complex and less predictable, and the entire sequence may take an extended period of time to complete. The purpose of the present study was to explore whether working memory also plays a role in a more ecologically valid instruction-following task designed to mimic the everyday practical demands imposed on children in their school life. This was captured by a 2-D computer-simulated environment of a school, in which the children received instructions to perform sequences of actions either within a single classroom or through navigation across multiple locations in a virtual school building.

Following instructions through to successful completion requires simultaneously holding in mind the detailed content of the sequence while monitoring ongoing performance. This capacity to maintain information while engaged in other cognitive activities is a key feature of working memory (e.g., Baddeley, 2012; Cowan, 2005; Oberauer, 2002, 2013; Shipstead, Lindsey, Marshall, & Engle, 2014). There are many alternative theoretical accounts of working memory, but a common feature shared across models is that working memory involves limited-capacity storage combined with attentional control (e.g., Cowan, 2005; Luck & Vogel, 2013; Oberauer, 2002; Oberauer, Süß, Wilhelm, & Wittman, 2003; Shipstead et al., 2014). The multiple-component model, introduced by Baddeley and Hitch (1974) and later revised by Baddeley (2000, 2012), has been particularly valuable in advancing our understanding of how individuals follow instructions. This consists of a central executive responsible for attentional control within and beyond working memory, which is supported by two specialized limited-capacity stores—the phonological loop and the visuospatial sketchpad—that are responsible for the maintenance of verbal and visuospatial information, respectively. The capacity of the two domain-specific slave systems is typically assessed using short-term memory measures involving the simple storage and retrieval of information, whereas the domain-general resources of the central executive are measured using complex working memory tasks comprising concurrent processing and storage of material (see, e.g., Conway et al., 2005). The newest addition to the model, the episodic buffer, is a temporary multidimensional store that forms an interface between the subsystems of working memory and long-term memory (Baddeley, 2000).

Recent studies have identified a role for working memory in following instructions. Concurrent tasks designed to interfere with the central executive, phonological loop, and visuospatial sketchpad have been shown to disrupt the ability to follow written instructions (Yang et al., 2014). These results suggest that verbal instructions may be held in the phonological loop and supplemented by additional visuospatial information in the environment, with the central executive coordinating the execution of actions through the retrieval of information from these stores. In childhood, working memory provides crucial support for the retention of both activity-specific and classroom management instructions at school (Gathercole & Alloway, 2008; Gathercole, Lamont, & Alloway, 2006). Consistent with this, verbal complex memory span measures associated with the attentional-control aspect of working memory are closely linked with children’s abilities to perform task instructions such as *Pick up the yellow ruler and then touch the blue folder* (Engle et al., 1991; Gathercole et al., 2008).

The tasks in this area of research (Allen & Waterman, 2015; Engle et al., 1991; Gathercole et al., 2008; Yang et al., 2015b; Yang et al., 2015a; Yang et al., 2014) typically involve immediate implementation of lengthy instructions. In this respect, they do not capture the prolonged-retention element that is intrinsic to day-to-day situations, which frequently involve remembering the later steps of an instruction sequence for up to several minutes while earlier actions are completed in turn. When the time needed to complete an extended ongoing sequence exceeds the temporal duration of working memory (estimated at between 2 and 18 s; e.g., Baddeley & Scott, 1971; Brown, 1958; Cowan, Saults, & Nugent, 1997; Peterson & Peterson, 1959), the rememberer must actively maintain the instructions in working memory, either through rehearsal to prevent decay, or through reactivation of memory traces by rapid switching of attention (e.g., Camos, Lagner, & Barrouillet, 2009; Cowan, 2005; Johnson, 1992). Storage of information could also be supplemented by long-term episodic memory, but because it is less accurate at retaining verbatim than gist information (e.g., Cowan, 2008; Pause et al., 2013; Tulving, 1983, 2002), it may not be as effective as working memory at preserving the literal content needed to follow relatively arbitrary instructions.

In order to explore the potential role of working memory in more complex situations that vary the amount of time over which sequences of instructions must be retained, we developed two new virtual versions of an existing following-instructions paradigm (Gathercole et al., 2008). Using a 2-D virtual environment enabled us to present ecologically salient stimuli in a computer-simulated setting that is meaningful and familiar, combining the advantages of a naturalistic paradigm with the appropriate degree of experimental control (e.g., Logie, Trawley, & Law, 2011; Rajendran et al., 2011; Rizzo & Buckwalter, 1997). Both tasks required participants to navigate through a virtual school environment to perform a sequence of spoken instructions. In one task, participants carried out a series of instructions on objects that were laid out on tables positioned around the edges of a single classroom (e.g., *Pick up the yellow folder and then touch the green bottle*). This task was similar to the original paradigm developed by Gathercole and colleagues (e.g., *Touch the red pencil and then pick up the blue box*), except that the visual environment was digital rather than in the physical world, and the to-be-manipulated items were distributed across different desks in the digital room rather than located on a single real desk within easy reach. In the second task, participants moved between multiple rooms in a virtual school to implement instructions (e.g., *Go to the IT Suite and touch the red ball and then go to Mrs. Bolton’s room and pick up the green pens*). In this way, the additional navigation demands of these activities increased in complexity (i.e., from the real-world task to a single-room virtual task to a multiroom virtual task), aligning the tasks more closely with the more complex situations in which children follow multistep instructions in school. Measures of verbal and visuospatial short-term memory and working memory, as well as the original real-world following-instructions task (Gathercole et al., 2008), were also administered.

This study was exploratory in nature and aimed to address two specific issues. First, we investigated the working memory processes underlying performance on the virtual following-instructions task. Recent studies have demonstrated that verbal working memory ability is associated with the accuracy with which children followed spoken instructions involving sequences of actions (Engle et al., 1991; Gathercole et al., 2008; Jaroslawska, Gathercole, Allen, & Holmes, 2015). Here we asked whether instruction-following involves the same processes in the virtual environment as in the real-world task. Second, we explored the links between the individual subcomponents of working memory and following-instructions task performance. We sought to establish whether the virtual tasks depend on the central executive, phonological loop, and visuospatial sketchpad in the same way as they do in the real-world paradigm (Yang et al., 2014). More specifically, we investigated the strength of links between individual differences in these aspects of working memory and performance on a task requiring immediate recall with no navigational demands (i.e., the real-world paradigm), on a task requiring immediate recall with minimal navigation load (i.e., the single-room virtual task), and on a task involving extensive navigation and reduced opportunities for motor planning (i.e., the multiroom virtual task). One possibility is that the distinctive 2-D-to-3-D mapping required in navigation of the virtual environment might induce significant visuospatial working memory involvement. Evidence to date suggests that in contrast, the visuospatial storage demands of instruction-following in real-world tasks are minimal (Jaroslawska et al., 2015). The importance of this research is to establish whether working memory plays a role in situations more typical of everyday instruction-following scenarios. Evidence for the involvement of working memory in the new virtual tasks would also provide an excellent starting point for developing practical assessments of working memory with high face validity that could be of great use to clinical and educational practitioners seeking tools to identify individuals with memory problems.

## Method

### Participants

A total of 42 children (21 boys, 21 girls) attending a primary school in the Southeast of England participated in the study. The mean age of the sample was 9 years 4 months (*SD* = 11.66 months; minimum = 7 years 10 months, maximum = 11 years 1 month).

### Procedure

Children were assessed individually in a quiet area of the school, seated at a table opposite the experimenter or in front of a laptop PC. Testing took place across two separate sessions, with each session lasting approximately 60 min. Eight subtests from the Automated Working Memory Assessment (AWMA; Alloway, 2007) and a real-world manual following-instructions task with 3-D props (Gathercole et al., 2008) were administered in the first testing session, with the order of the tasks randomized across participants. Two virtual following-instructions tasks were completed in the second session. The order of these tasks was fixed so that the single-classroom version of the task always preceded the more complex multilocation version. Written parental consent was obtained prior to testing. The study was approved and conducted in accordance with the guidelines of the Cambridge University Psychology Research Ethics Committee and the MRC Cognition and Brain Sciences Unit.

### Materials

#### Real-world following-instructions task

Participants were required to memorize and carry out sequences of action commands on an array of concrete, 3-D props. The objects were a set of five stationery items (a ruler, an eraser, a pencil, a folder, and a box) in each of three colors (red, yellow, or blue). Two actions could be performed, touch (e.g., *touch the red pencil*) and pick up (e.g., *pick up the yellow ruler*), which were concatenated using the adverb “then” to produce increasingly longer sequences of instructions that varied in length but not in grammatical complexity. The items used in each instruction were selected at random, with the constraints that no repetition of color and object combinations occurred in the instructions as a whole.

The objects were positioned randomly on a desk within arm’s reach of the child. Prior to testing, the children were asked to name and identify all objects and their labels to ensure that they knew what the objects were. The instruction sequences were read aloud by the experimenter at a measured rate. A span-type procedure was employed in which the length of the instruction sequence increased systematically. Each span consisted of a block of six trials. Testing started at one action (e.g., *Touch the red ruler*), increased by one action per block (e.g., *Touch the red ruler and then pick up the yellow pencil*), and was terminated after three incorrect trials in one block. The object array was in view at all times. Participants listened to the instructions and were restricted from manipulating any of the objects. At the end of the presentation, participants were asked to perform the actions in sequence. Responses were recorded as accurate if all elements of the individual action phrases—action, object, and color—were correctly recalled in their original serial position in the instruction sequence.

#### Virtual following-instructions tasks

These tasks required participants to follow instructions in two computerized environments: a single classroom, and a whole school environment with multiple rooms. Both tasks were created using the Source Software Development Kit (Source SDK) supplied with the video game Half-Life 2 (see Trawley, Law, Logie, & Logie, 2011, for a detailed case study using the Source 3-D game engine). The Valve Hammer Editor was used to construct an on-screen 3-D school building. The tasks were viewed on a 15-in. color monitor and run on a Dell laptop with an Intel Core i5 processor and Intel HD Graphics 4000 graphics card. To facilitate the data analysis, a data extraction utility was created with the Python programming language.

Participants navigated around the virtual environment using the keyboard and mouse. The keyboard was used for forward, lateral, and backward movements (“↑,” “→” or “←,” and “↓” keys, respectively) and for the physical actions “touch” and “pick up” (the “t” and “p” keys, respectively). The mouse provided control over visual pitch (up and down) and yaw (spin left and right) perspectives. All actions made by the participants were automatically recorded by the software.

A span-type procedure was employed in both tasks, in which the number of to-be-remembered elements was increased over successive trials until the discontinue rule was met. Each span consisted of a block of six trials. Testing started at one action (e.g., *Touch the red ball*), increased by one action per block (e.g., *Touch the red ball and then pick up the yellow folder*), and was terminated after three incorrect trials in one block. Performance accuracy was scored according to a strict recall criterion in which all individual elements of the instruction sequence had to be performed in their correct serial order to be scored as correct.

Prior to any testing, all participants completed two practice sessions: one for the objects and actions used in the paradigm, and another for locations. These sessions were designed to familiarize the participants with the aim of the task and the mouse and keyboard controls for moving around the environment. In each of the five trials of object–action practice, the participants first heard a sample action phrase over headphones (e.g., *Pick up the yellow folder*) and were required to carry it out by walking up to the specified object using the navigation keys and then pressing the correct action key on the keyboard (i.e., “t” for touch or “p” for pick up). Visual feedback, in the form of a green tick mark or a red cross, was provided after every response. All five trials had to be performed correctly in order to complete the object–action session (erroneous responses had to be revised, repeatedly if necessary). During location practice, the participants explored the virtual school environment with the help of the experimenter. They were instructed first to read out loud the signs displayed outside each door and then to enter each room in turn. Location practice was completed once all rooms had been labeled and entered. In addition to these initial practice sessions, participants completed two mock trials at the beginning of each task.

##### Single-room virtual task

**Fig. 1:**
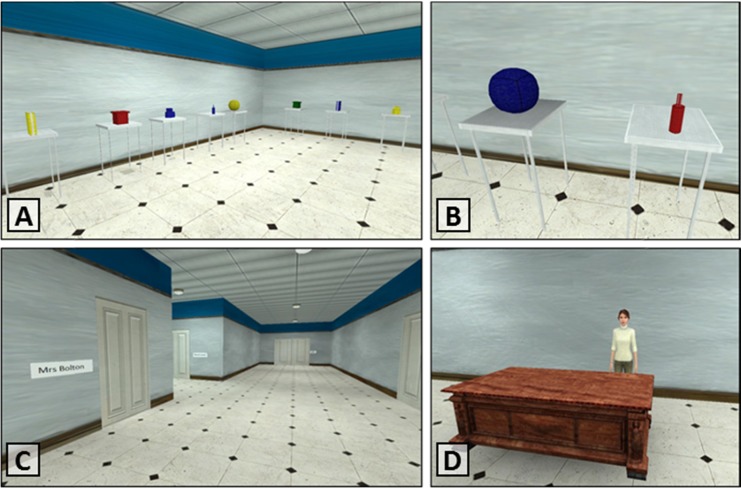
Screen shots of the virtual school: (A) the layout of the items in one of the classrooms, (B) a close-up view of sample objects, (C) the main corridor of the virtual school building, and (D) the head teacher’s office.

The single-classroom task was an on-screen version of the 3-D manual following-instructions paradigm developed by Gathercole et al. (2008). At the start of each trial, the participant was presented with a first-person view of a classroom, with desks laid out around the edges of the room (see Fig. 1). Different items of stationery stood on each desk. Participants received spoken instructions over headphones, which they were then required to carry out in serial order by walking up to the objects with the navigation keys and then pressing the correct action key on the keyboard (i.e., “t” or “p”). The stimuli and action phrases were constructed in the same way as those in the real-world task: There were five objects (a ball, a folder, a box, pens, and a bottle) in each of four colors (green, red, yellow, and blue) and two actions (touch and pick up); the instruction phrases were concatenated using the adverb “then” in order to produce sequences that varied in length but were not linguistically complex. The items used in each instruction were selected at random, with the constraint that no repetition of color and object combinations occurred in the instructions as a whole. Seeing one action-based command through to completion could take between 6 and 12 s, depending on the exact location of the object. No time restrictions were imposed on the participants during testing.

##### Multiroom virtual task

This task was similar to the single-room version, but with the addition of multiple locations around the school building. Participants began each trial in the head teacher’s office, where they heard a spoken instruction sequence. To complete the sequence, participants were required to use the keyboard to navigate through different locations around the virtual school. Seven different locations were connected by three corridors (i.e., Mr. Taylor’s room, Mrs. Lloyd’s room, Mr. Lewis’s room, Mrs. Bolton’s room, Hall, IT Suite, and Stockroom). The room dimensions and the layout of the items were identical in each location. Figure 1 shows screenshots of the task taken from a subset of locations. The locations and items used in each instruction were selected at random, with the constraint that no repetition of rooms or color and object combinations occurred in the instructions as a whole. Reaching different locations (i.e., navigating from the head teacher’s office to a particular classroom) could take between 15 and 25 s, depending on the exact distance between the starting point and the target location. No time restrictions were imposed, and participants were free to enter and reenter the rooms at all times.

#### Automated Working Memory Assessment (AWMA)

Eight standardized subtests from the AWMA (Alloway, 2007) were administered. These included two tests each of verbal short-term memory (digit recall and nonword recall), visuospatial short-term memory (dot matrix and block recall), verbal working memory (listening recall and backward digit recall), and visuospatial working memory (Mr. X and spatial recall).

The verbal short-term tests required the immediate serial recall of verbal stimuli (digits or nonwords). For the visuospatial short-term memory tasks, participants were required to recall in serial order a sequence of dots in a 4 × 4 matrix (dot matrix) or a sequence of locations tapped out on 3-D blocks (block recall). Participants were required to recall a series of digits in reverse serial order for the backward digit recall task. In the listening recall test, children verified whether a series of sentences were factually true or false, before recalling the final word of each of the sentences in the order in which they had heard them. For the Mr. X task, participants were asked to decide whether two figures presented on screen were holding a ball in the same hand as one another. The ball held by the figure on the right could appear at one of six possible compass points. Having decided whether the two figures were holding the ball in the same hand, participants were then asked to recall the location of the ball held by the figure on the right. In the spatial recall test, pairs of identical shapes were presented on screen. The shape on the right had a dot on it and appeared in one of seven rotated positions. Participants were asked to judge whether the shape on the right was the same as or was the mirror-image of the shape on the left before recalling the location of the red dot at one of three possible positions.

Trials were presented in blocks of six. Each task started at a span of one item (except for backward digit recall, which started at two items) and increased in length by one item in each subsequent block. Every correct trial was scored as 1. If a child responded correctly to the first four trials within a block, the program automatically proceeded to the next block (i.e., next span level). If three errors were made within a block, the task discontinued. Raw scores were reported for all tests to enable the analysis of age-related changes in instruction-following. In addition, *z* scores were computed for each variable and averaged to provide a composite score for each of the four aspects of working memory. The standardized working memory scores for the sample are reported in Table 1. The mean values were in the high average range, with reasonable degrees of variability around the group mean (*SD*s between 10 and 17).

**Table 1:** Standard working memory scores

	Short-Term Memory				Working Memory			
	Verbal		Visuospatial		Verbal		Visuospatial	
	1	2	3	4	5	6	7	8
Mean	119.64	99.75	111.76	107.02	109.21	110.69	107.81	111.52
*SD*	16.53	12.22	11.64	10.96	13.43	13.66	16.90	13.22
Skewness	–0.48	–0.91	–0.45	–0.22	0.84	0.38	–0.54	–0.03
Kurtosis	–0.15	0.17	0.07	0.85	0.24	0.64	0.43	–0.36

The numbers at the top correspond to 1 *digit recall*, 2 *nonword recall*, 3 *dot matrix*, 4 *block recall*, 5 *listening recall*, 6 *backward digit recall*, 7 *Mr. X*, and 8 *spatial recall* tests.

## Results

Descriptive statistics for the working memory assessments and all three following-instructions tasks are provided in Table 2. Raw scores on the three following-instructions tasks increased with age. A correlation between age and task scores accordingly established that older children performed significantly better on all three following-instructions paradigms [*r*(42) = .478, *p* < .01, for the manual task; *r*(42) = .388, *p* < .05, for the single-room task; and *r*(42) = .335, *p* < .05, for the multiroom virtual task]. Recall accuracy was highest in the real-world task and lowest in the multiroom virtual task. There were significant associations between all three instruction-based tasks. When controlling for age, both versions of the computerized following-instructions task correlated with the original manual task developed by Gathercole et al. (2008): *r*(39) = .572, *p* < .001, for the single-room task, and *r*(39) = .568, *p* < .001, for the multiroom task. The association between the two virtual tasks was also highly significant, *r*(39) = .719, *p* < .001.

**Table 2:** Raw scores for working memory measures and following-instructions tasks

	Total (*N* = 42)		Year 3 (*N* = 15)		Year 4 (*N* = 11)		Year 5 (*N* = 16)	
	Mean	*SD*	Mean	*SD*	Mean	*SD*	Mean	*SD*
Working Memory Measures								
Verbal Short-Term Memory								
Digit recall	32.52	5.25	30.93	5.05	33.09	3.39	33.63	6.31
Nonword recall	12.00	3.89	10.67	3.96	11.09	4.25	13.88	2.92
Visuospatial Short-Term Memory								
Dot matrix	24.98	4.13	22.53	2.29	23.45	4.13	28.31	3.28
Block recall	24.50	4.49	21.47	3.58	24.18	3.22	27.56	4.11
Verbal Working Memory								
Listening recall	13.17	3.75	10.87	2.39	13.73	3.90	14.94	3.77
Backward digit recall	15.50	4.95	12.47	2.26	17.09	5.32	17.25	5.40
Visuospatial Working Memory								
Mr. X	13.19	5.75	10.47	4.44	13.09	4.61	15.81	6.55
Spatial recall	19.86	5.91	17.87	4.41	17.82	5.23	23.13	6.39
Following-Instructions Tasks								
Real-World Task	12.93	2.41	11.66	2.50	12.36	1.57	14.50	2.00
Virtual Task								
Single-room	8.86	3.63	7.40	3.07	7.91	3.45	10.88	3.48
Multiroom	5.33	1.91	4.67	1.76	4.73	1.62	6.38	1.86
Age (in Months)	110.52	11.51	98.00	2.83	109.55	4.11	122.94	4.65

Correlations between working memory and performance on the instructions tasks, controlling for age, are presented in Table 3. Significant associations were revealed between performance on the real-world following-instructions task and both the digit and nonword recall scores, as well as the composite verbal short-term memory measure. Recall accuracy in the single-room virtual task was related to both measures of verbal short-term memory—nonword recall and digit recall—and to the corresponding verbal short-term memory composite score. Scores on this task were also linked to performance on the listening recall task and the composite verbal working memory score. Children’s performance on the multiroom virtual environment task was related to digit recall and to both the verbal short-term and verbal working memory composite scores. In all cases the correlations were positive: Higher scores on the instruction-following tasks were associated with better performance on the working memory tasks. The only significant association related to visuospatial aspects of working memory emerged between the real-world paradigm and Mr. X.

**Table 3:** Correlations between working memory measures and performance on following-instructions tasks, controlling for age

	Following-Instructions Task		
		Virtual	
	Real-World	Single-Room	Multiroom
Working Memory Measures			
Verbal Short-Term Memory			
Digit recall	.470^**^	.451^**^	.496^**^
Nonword recall	.316^*^	.346^*^	.292
Composite	.447^**^	.453^**^	.449^**^
Visuospatial Short-Term Memory			
Dot matrix	.158	.055	.192
Block recall	.136	.021	.248
Composite	.174	.045	.261
Verbal Working Memory			
Listening recall	.285	.349^*^	.304
Backward digit recall	.182	.249	.277
Composite	.264	.338^*^	.330^*^
Visuospatial Working Memory			
Mr. X	.327^*^	.145	.180
Spatial recall	.113	.149	.289
Composite	.250	.167	.266
Working Memory Factor Scores			
Factor 1	.097	–.009	.163
Factor 2	.399^*^	.457^**^	.403^**^

^*^ *p* < .05, ^**^ *p* < .01.

To provide reliable and robust indices of performance on the working memory tasks, an exploratory factor analysis with Varimax rotation was performed on the raw AWMA (Alloway, 2007) scores. Although the sample size was relatively small, the subject-to-variable ratio was larger than 5:1 (e.g., Arrindell & van der Ende, 1985; Bryant & Yarnold, 1995; MacCallum & Widaman, 1999), and the analysis produced a clear and interpretable factor structure. Two factors emerged with eigenvalues in excess of 1.00, explaining 51.34 % and 16.43 % of variance, respectively. Factor loadings greater than .30 on the rotated factor matrix are shown in bold in Table 4.

**Table 4:** Rotated component matrix

Working Memory Measures	Factor 1	Factor 2
Digit recall	.088	**.890**
Nonword recall	.121	**.782**
Dot matrix	**.802**	.182
Block recall	**.783**	.233
Listening recall	**.398**	**.712**
Backward digit recall	**.463**	**.640**
Mr. X	**.771**	.246
Spatial recall	**.832**	.129

Factor loadings over .3 are marked in bold.

All four visuospatial memory measures loaded highly on Factor 1, whereas the four verbal measures loaded most highly on Factor 2. Backward digit recall and listening recall loaded on both factors, although most strongly on the verbal factor. The first factor is therefore associated with visuospatial memory, and the second with verbal memory. This domain-specific factor structure mirrors those observed in previous studies using AWMA (e.g., Jarvis & Gathercole, 2003). The verbal factor correlated strongly with all three following-instructions tasks: *r*(39) = .399, *p* < .05, for the real-world task; *r*(39) = .457, *p* < .01, for the single-room virtual task; and *r*(39) = .403, *p* < .01, for the multiroom task. We found no significant links between Factor 1 (visuospatial memory) and instruction-following.

## Discussion

In a newly developed virtual school environment designed to mimic the everyday classroom demands of instruction-following, children’s abilities to carry out sequences of actions following spoken instructions were closely related to their verbal working memory skills. Children with higher verbal memory scores were able to perform longer instruction sequences in two separate tasks, one involving performing instructions such as *Pick up the red folder and then touch the blue pens* in a single virtual room in which all objects were in the line of vision, and the other requiring navigation across different parts of the school that were unseen but with which the child was already familiar, such as *Go to Mr. Taylor’s room and touch the yellow box, and then go to the IT Suite and pick up the red folder*. These children were also superior in performing simple sequences of actions on a set of props laid out before them, such as *Pick up the blue ruler and then touch the yellow pencil*. In all three tasks, instruction-following abilities were unrelated to visuospatial aspects of working memory.

These findings build directly on earlier observations that children with low working memory scores struggle to follow instructions in everyday classroom situations (Gathercole, Lamont, & Alloway, 2006). They also extend the evidence linking individual differences in working memory and instruction-following to more ecologically valid contexts requiring complex navigation. To date, working memory involvement in following instructions has relied on artificial experimental paradigms requiring the manipulation of sequences of physical objects and symbols. For instance, in dual-task studies with adult participants, Yang, Allen, and Gathercole (2015) found performance of sequences of manual actions following written instructions to be impaired by concurrent tasks known to disrupt the central executive and both domain-specific stores of the multicomponent model of working memory (Baddeley, 2000; Baddeley & Hitch, 1974). Individual-differences studies have also established that children’s working memory spans are closely associated with their abilities to implement task instructions to complete pencil-and-paper tasks (e.g., *Point to the picture at the top of page threeand copy it twice*), carry out action-oriented tasks (e.g., *Sit on the floor Indian style*), and manipulate sequences of objects placed in front of them (e.g., *Pick up the green eraser and put it in the white bag*; Engle et al., 1991; Gathercole et al., 2008).

The present findings indicate that the verbal aspects of working memory involved in both simple and more complex span tasks played a highly specific role in the ability to follow spoken instructions, both when the instructions were to be performed with physical props and in the more complex context of the virtual school. This is consistent with evidence that both patients with acquired damage of verbal short-term memory and poor readers with verbal short-term memory deficits are impaired in performing sequences of physical actions to verbal instructions (Cohen-Mimran & Sapir, 2007; Plaza, Cohen, & Chevrie-Muller, 1997; Smith, Mann, & Shankweiler, 1986; Warrington & McCarthy, 1983). These findings are likely to reflect the storage demands of remembering the verbal instructions as the action sequence is performed. Given the extended duration of time over which the instructions had to be carried out, the maintenance of verbal information is likely to be supported by either verbal rehearsal in the phonological loop (Baddeley, 2000) or refreshing of memory traces through attentional focusing (e.g., Camos et al., 2009; Cowan, 2005; Hudjetz & Oberauer, 2007; Johnson, 1992). It is not possible to distinguish between these two mechanisms in the present data, but speculatively, subvocal rehearsal may support the maintenance of the entire verbal sequence, with attentional refreshing bringing the current step of the instructions into focus.

Visuospatial aspects of memory, on the other hand, were unrelated to performance on any of the three instruction-following tasks. This indicates that neither the manipulation of physical props nor unfamiliar key-based navigation of a virtual environment depends on simple storage of visuospatial coordinates. This finding is at odds with studies reporting close associations between spatial navigation around similar virtual environments and visuospatial working memory, but weak links with verbal working memory (Garden, Cornoldi, & Logie, 2002; Logie et al., 2011). These inconsistencies may reflect differences in the paradigms employed. Both Logie et al.’s virtual multiple-errands task and Garden et al.’s route-learning task required extensive spatial planning. In the multiple-errands task, participants learned a set of to-be-completed tasks and studied the layout of the virtual environment to work out the optimum order in which to carry out the tasks to complete them within a given time limit. Likewise, in the route-learning tasks participants were required to learn a spatial route for later retrieval. In contrast, substantially greater demands were placed on verbal sequence learning in the present virtual school tasks. Verbal instructions were presented only once and had to be carried out in serial order, which activated the phonological loop. Differences in the strength of the associations with working memory across studies may therefore reflect differences in the task demands, which promoted either spatial planning or verbal rehearsal.

It is possible that other aspects of working memory that were unmeasured in the present study may also have contributed to performance on these tasks. Other research using prop-based following-instructions paradigms has pointed to the possible existence of a motor store providing temporary maintenance of spatio-motoric representations of both planned and executed actions (see Allen & Waterman, 2015; Jaroslawska et al., 2015; Smyth & Pendleton, 1989, 1990). The capacity of this putative store may be closely related to the practical abilities to follow instructions. There may be greater involvement of such a store in real-world tasks involving gross motor gestures than in virtual tasks that require keypresses for actions, rather than motor actions per se. Future research will be needed to test this hypothesis.

In the present study, we demonstrated that computerized virtual environments can provide valuable paradigms for assessing complex cognition. The majority of cognitive assessments are hampered either by relying on real-world situations over which the experimenter has very little control, or by tightly controlled laboratory measures that are not well correlated with real-life situations (Alderman, Burgess, Knight, & Henman, 2003; Burgess et al., 2006; Cockburn, 1995; Kingstone, Smilek, Ristic, Friesen, & Eastwood, 2003). The virtual following-instructions tasks described here tap into one of the ways in which children use working memory in the classroom, and this study provides important new evidence validating these tasks against more traditional laboratory-based tests of verbal working memory. They might therefore provide a good starting point for developing ecologically valid assessments of working memory for teachers and other educational professionals who are seeking to identify children with poor working memory who may be at risk of slow academic progress (Gathercole & Alloway, 2008). These tasks might also offer a valuable way of embedding working memory training in activities that more closely resemble working memory’s everyday uses. To date, intensive adaptive working memory training has been successful in boosting performance on untrained working memory tasks with task demands similar to the training activities, but there is little reliable evidence that it enhances performance on more practical everyday tasks (e.g., Dunning, Holmes, & Gathercole, 2013). To overcome this very narrow window of transfer, the gap between more artificial training programs and everyday situations that depend on working memory needs to be closed (Redick, Shipstead, Wiemers, Melby-Lervåg, & Hulme, 2015). The virtual tasks developed here have potential for the delivery of training in the context of following instructions, a situation corresponding closely to children’s everyday use of working memory in the classroom.
